# USP14 as a Therapeutic Target Against Neurodegeneration: A Rat Brain Perspective

**DOI:** 10.3389/fcell.2020.00727

**Published:** 2020-07-31

**Authors:** Chayan Banerjee, Moumita Roy, Rupsha Mondal, Joy Chakraborty

**Affiliations:** ^1^Department of Cell Biology and Physiology, CSIR-Indian Institute of Chemical Biology-TRUE, Kolkata, India; ^2^CSIR-Indian Institute of Chemical Biology, Kolkata, India; ^3^Academy of Scientific and Innovative Research (AcSIR), Ghaziabad, India

**Keywords:** USP14, Prohibitin2, mitophagy, neurodegeneration, Parkinson’s disease, substantia nigra, rotenone, 3-nitropropionic acid

## Abstract

In the recent past, many of the deubiquitinases (DUB) were found to modulate mitochondrial clearance or mitophagy and thus they are currently projected as therapeutic targets against neurodegeneration. Among these DUBs, USP14 stands at a distinctive juncture, since it can influence both proteasome complex activity and autophagy process. USP14 interference can enhance mitochondrial clearance and thus can protect Parkinsonian phenotypes in *Drosophila* model. However, in higher animal models of neurodegenerative disorders, evaluation of the protective role of USP14 is yet to be done. In this perspective, we pointed out a few of the major considerations that should be classified before designing experiments to evaluate the therapeutic potential of this DUB in rodent models of neurodegeneration. These are mainly: level of USP14 in the concerned brain region and how the level alters in the model system. Because USP14 mediated mitophagy is Prohibitin2 dependent, the anticipated impact of this protein in this aspect is also discussed. To illustrate our view, we show that USP14 levels increases in adult rat brain substantia nigra (SN) and cerebellum compared to the young ones. We also depict that rotenone treatment can immediately lead to increased SN specific USP14 levels. Our perception thus portrays USP14 as a therapeutic target, especially for addressing SN specific neurodegeneration in adult rat brain, but may vary with the disease model.

## Introduction

Dysfunctional mitochondria can lead to Cytochrome c release in cytosol and thus multiple levels of adjustments are required to maintain a healthy mitochondrial population in a normal cell. While doing so, the damaged ones are degraded by a process called mitochondrial autophagy or mitophagy. Other than autophagic machineries, the process also depends on ubiquitin proteasome system (UPS) to some extent (Tanaka et al., 2010; Chan et al., 2011; Yoshii et al., 2011). The current understanding of this dependency suggests that UPS degrades outer mitochondrial membrane (OMM) proteins and thus exposes inner mitochondrial membrane (IMM) LC3 receptor – Prohibitin2 (PHB2) (Wei et al., 2017). This facilitates the process of engulfment by autophagic isolation membranes. During mitophagy, UPS degrades the OMM proteins which are targeted by a few E3 ubiquitin ligases; among them, Parkin is the most studied one. Activation of Parkin and recruitment onto depolarised mitochondria is facilitated by PINK1 (a kinase) and this pathway has been studied with great details (Narendra et al., 2008, 2010; Ziviani et al., 2010; Chakraborty et al., 2017). Mutations in PINK1 and Parkin have been directly linked to familial form of Parkinson’s disease (PD) (Kitada et al., 1998; Silvestri et al., 2005). Though most of the cases are sporadic, anomaly in the maintenance of a healthy mitochondrial population is equivocally accepted in both genetic and sporadic forms of the disorder (Parker et al., 1989; Mizuno et al., 1998). In this disorder, dopaminergic neurons at substantia nigra (SN) region of the brain progressively degenerate. The manifestation at the periphery includes tremor, akinesia and rigidity. Cure of the ailment involves dopamine supplementation but comes with severe side effects and the current therapies do not halt or slow down neurodegeneration. However, huge efforts are made to delineate the cause for the region specificity in PD and strategies are designed to make the hit points “druggable.” Among these, recent discoveries indicate that deubiquitinase enzymes (DUBs) might have a higher impact on the development and progression of neurodegeneration. Here, we discuss how DUBs are important for PD and a few key aspects that should be considered before evaluating USP14 as a mediator of mitophagy in PD animal models.

## Deubiquitinases in Parkinson’s Disease: a Brief Description

As the name suggests, these enzymes cleave ubiquitin chains from the substrate. There are more than one hundred DUBs, involved in numerous pathways and they have some sort of substrate specificity, which makes them targets for drug development (Komander et al., 2009). As far as PD is concerned, a few DUBs have been implicated in the progression of the disease, both in cellular and animal models. The idea in principle suggests that where efficient Parkin mediated ubiquitination is compromised, inhibiting DUBs might linger the remaining signal which originates from the other routes of mitophagy (SIAH, Mul1, Gp78, etc.). In general, these DUBs influence the disease scenario by antagonizing Parkin activity or by modulating UPS and autophagy. In this regard, USP15 and USP30 were found to antagonize Parkin activity by competing for the common substrates on OMM (Bingol et al., 2014; Cornelissen et al., 2014). Downregulation of both of these DUBs delivered protective effects against PD progression in *Drosophila* model. USP8 on the other hand regulates Parkin activation and downregulation of which is also known to be protective in *Drosophila* model of PD (Durcan et al., 2014; Von Stockum et al., 2019). Another DUB- Ataxin 3 has huge potential to be a drug target as it can directly interact with Parkin, but the impact on PD is yet to be documented (Durcan et al., 2012). Mutation of UCH L-1 in this respect is the only DUB directly linked with familial PD (Kabuta et al., 2008). It can inhibit autophagy by interacting with LAMP-2A. USP24 is another modulator of autophagy which may also influence PD progression as it can regulate dopaminergic neurite outgrowth, but the potential as a disease modulator is not evaluated yet (Li et al., 2006; Haugarvoll et al., 2009).

Among the DUBs, USP14 is unique as it can influence autophagy (Xu et al., 2016) and UPS activity (Lee et al., 2010; Kim and Goldberg, 2017) independently, both are the prerequisites for mitophagy. Recently, it was demonstrated that USP14 inhibition enhances mitophagy and protects against the disease progression in *Drosophila* model (Chakraborty et al., 2018). So the current understanding depicts that higher levels of USP14 may influence UPS activity, autophagy, and in turn mitophagy levels. All of these processes are affected during many of the age-related neurodegenerative disorders, including PD. Verification of the protective ability of USP14 inhibition in higher animal models of neurodegeneration is yet to be done. The lone report which supports this potential of USP14 inhibition is done in cerebral ischemia/reperfusion-induced neuronal damage model (Min et al., 2017). None the less, this study indicates that the inhibitor of USP14 – IU1 might be blood brain barrier permeable. In this perspective, we highlighted a few of the aspects that should be considered for better interpretation of data, as far as USP14 vs. neurodegeneration is concerned.

## USP14 vs. Neurodegeneration: Considerations for Extrapolation

Inhibitors of USP14 are available. A popularly used inhibitor – IU1 increases UPS activity, enhances Tau degradation in primary cultured neurons and increases mitochondrial elimination in neuronal cell lines (Lee et al., 2010; Boselli et al., 2017; Chakraborty et al., 2018). For evaluating the potential of USP14 inhibitor in rodent models of PD, here we discuss some of the key factors that might influence the outcome of the experiments.

**FIGURE 1:**
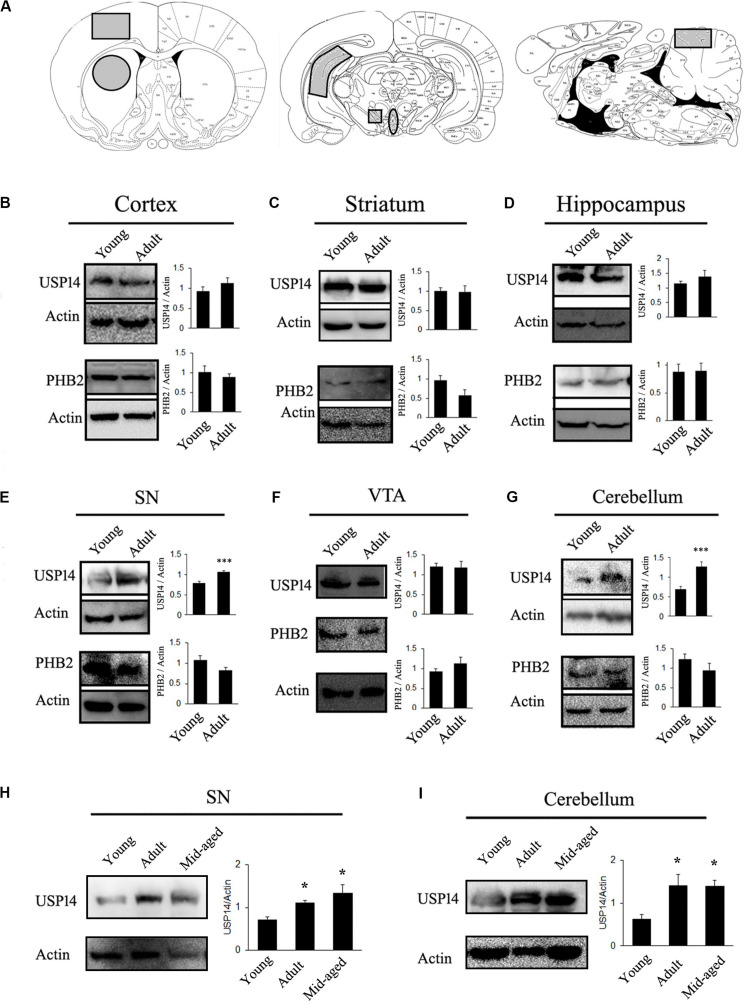
USP14 increases with age in substantia nigra and cerebellum. **(A)** Different brain regions showing the area that were taken for the study (marked as gray). **(B–G)** Immunoblot analysis of USP14, PHB2 and Actin from the mentioned area of brain from young (1 month) and adult (6–7 months) animals. **(H,I)** Immunoblot analysis of USP14 from the mentioned areas of young (1 month), adult (6–7 months) and mid-aged (10–12 months) rats. Bar graphs represent mean ± SEM. *N* = 8 for **(B–G)** and 3 for **(H,I)**. We employed student’s *t* test for **(B,G)** and one way ANOVA followed by Newman Keul’s multiple comparison test for statistical significance for **(H,I)**. *p ≤ 0.05, ***p ≤ 0.001.

First of all, level of USP14 in a brain region or subset of neurons may vary during aging. If this hypothesis is true, the amount of USP14 inhibition required for neuroprotection might change with the age of the model. In other words, if a neurodegenerative disease model can be generated using animals from different age groups, level of USP14 should be monitored in that particular age group to adjust the dose of the inhibitor. To elaborate our opinions, first we selected rats from two age groups (Sprague-Dawley, young −1 month and adult- 6 to7 months old) and compared USP14 levels at different brain regions. We found that USP14 is increased in adult rat brain SN and cerebellum (Figures 1E,G), while there were no significant alterations in cortex, striatum, VTA and hippocampus (Figures 1A–D,F). Accessibility and distribution of USP14 inhibitors among these brain regions are yet to be characterized. Obviously the level of the protein and the accessibility of the inhibitor in that region should equally complement each other. Most commonly, while developing PD models with rotenone, adult rat is preferred as the young ones often show mild behavioral or neuroanatomical changes in response to acute neurotoxic insults (Cannon et al., 2009). Our results are indicative that changes in USP14 levels might make a brain region more vulnerable than the others (Figure 1E). However, further independent investigations are required to press upon this point and decipher whether or not other UPS related DUBs compensate this effect. We further wanted to quantify if this increase in USP14 is maintained in older rats or it reverses back. So we included another age group (10–12 months) to further investigate. We found that the increase in USP14 was maintained in mid-aged rat brain SN and cerebellum (Figures 1H,I).

Secondly, if evaluation of USP14 as a therapeutic agent is connected with mitophagy, it should be kept in mind that mitophagy, in general, may depend to a certain extent on PHB2 (Wei et al., 2017) and USP14 mediated mitophagy is PHB2 dependent (Chakraborty et al., 2018). However, PHB2 levels should not be considered directly as an indicator of neuron’s ability to thrust for mitophagy. PHB2 mediated mitophagy is mostly UPS dependent and there are different pathways that can still drive mitophagy in response to diverse cellular stimuli. Though many of the functions of PHB1 and 2 are well documented (Merkwirth et al., 2008), their brain region-specific distribution is not fully classified yet. Also, the correlation between USP14 and PHB2 level is yet to be deciphered. However, it can be envisioned that if a group of neurons express a very low amount of PHB2, USP14 inhibition might not be able to enhance mitophagy significantly and may lead to unpredictable changes at the cellular level. We found that PHB2 levels do not alter due to age in the mentioned areas of rat brain (Figure 1).

The third major point that should be considered is the process of the model generation. Some models of neurodegenerative disorders show protein aggregate formation at some point in time and those aggregates may block UPS functionality (Bence et al., 2001). Unless there is formation of unoccupied proteasome complex, USP14 blockade might not be able to execute the desired effect. So, the time point from when the inhibitor is administered is something vital to consider, preferably before the formation of the protein aggregates. Many of the animal models of neurodegeneration are generated by the use of mitochondrial electron transport chain (ETC) complex inhibitors, like rotenone (Sherer et al., 2003; Cannon et al., 2009), 1-methyl-4-phenyl-1,2,3,6-tetrahydropyridine (Burns et al., 1984; Heikkila et al., 1984) and 3-nitropropionic acid (3-NP) (Beal et al., 1993; Chakraborty et al., 2014a). How USP14 is modulated by these toxins should also be taken under consideration. To elaborate the hypothesis that different mitochondrial toxins may alter USP14 differently, even before the initiation of neurodegeneration, we selected two of the commonly used mitochondrial toxins, namely rotenone (Sherer et al., 2003; Cannon et al., 2009) and 3-NP (Beal et al., 1993; Pandey et al., 2008; Chakraborty et al., 2014a). One of the reasons to choose these two toxins was their ability to block mitochondrial ETC complex I (by rotenone) and II (by 3NP) ubiquitously. Rotenone is widely used to generate neurodegeneration in dopaminergic cells of SN (Sherer et al., 2003; Cannon et al., 2009) and 3-NP is used mostly for striatal lesions (Beal et al., 1993; Benchoua et al., 2008; Chakraborty et al., 2014a). Though 3-NP is also known to induce mild neuronal loss at SN (Fernagut et al., 2002), these toxins have different mechanisms to generate area-specific neurodegeneration. Along with SN, two major brain regions were selected: striatum and cerebellum. We also selected ventral tegmental area (VTA) because of its proximity and similarity with SN in terms of dopaminergic neuronal population.

**FIGURE 2:**
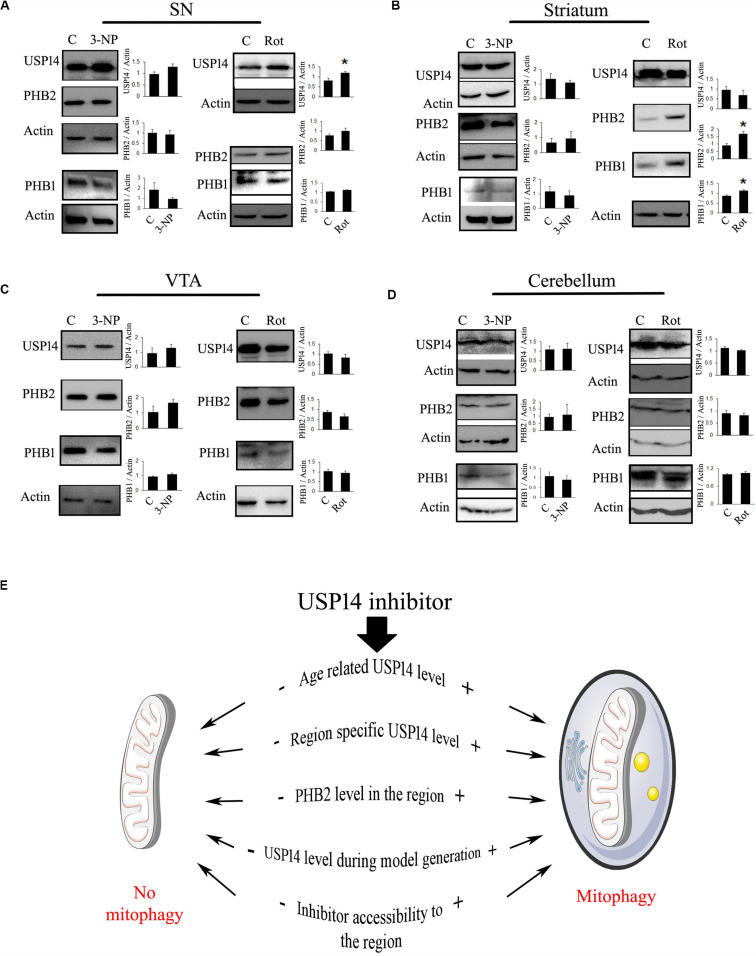
Blockade of mitochondrial electron transport chain complex I increase USP14 levels in substantia nigra. **(A–D)** 6–7 month old animals were treated with rotenone (1 mg / Kg, 2 days) or 3-NP (20 mg/Kg, 2 days) were sacrificed and brain regions were isolated. Immunoblot analysis was done for the mentioned areas and proteins. Bar graphs represent mean ± SEM from 3 to 4 animal brain. Student’s *t* test, *p ≤ 0.05. **(E)** Different possibilities to consider for USP14 inhibition mediated mitophagy. (+) and (–) depict increased or decreased levels of the event, respectively, before USP14 inhibitor administration and that might decide the outcome after. The list includes some of the possibilities and might not be exhaustive.

Previously it was found that 20 mg/kg dose of 3-NP starts showing neuronal lesions from 4th day of treatment, however, the initial behavioral symptom starts appearing from 3rd day onwards (Pandey et al., 2008; Chakraborty et al., 2014a, b). To monitor the effect of complex II inhibition on USP14 at the very early stage, before majority of the neurons are lost, we treated 3-NP for 2 days and the animals were sacrificed on the 3rd day. We did not find any change in USP14 levels after 3-NP administration in any of the mentioned brain regions (Figures 2A–D).

We wanted to monitor the immediate effect of rotenone treatment (1 mg/kg) on USP14 levels, as well. We found that rotenone treatment (1 mg/kg) did not show reduced dopamine levels upto 6th day of treatment, but starts showing weight loss, increased catalepsy and reduced rearing from 4th day (data not shown). So, we sacrificed the animals on 3rd day after two doses of rotenone. We found a significant increase in USP14 in SN after rotenone treatment, while no alteration in the other regions was observed (Figures 2A–D). As the effect of rotenone or 3-NP treatment on PHB1-PHB2 complex is not characterized yet, we also measured PHB1 along with PHB2 in these brain regions after these toxin treatments. Only striatum showed increased levels of PHB2, along with PHB1 after rotenone administration (Figure 2B).

From these two examples of mitochondrial ETC complex inhibitors, it is clear that different neurotoxins might offer differential alterations of USP14 (and PHB2) in different brain regions. It is always advisable to determine the level of USP14 in the respective brain regions of the animal model, before evaluating the prospects of USP14 inhibition. From these experiments it is not clear though whether the increase in USP14 is neuron-specific or not. Semi-quantitative immunofluorescence based co-localisation study is advised to delineate this point a step further. From these experiments, it is also not clear whether or not the increase in USP14 is accompanied by increased proteasome complex, along with other associated DUBs (USP14, UCH37, and RPN11) which are important for maintaining free ubiquitin pool. Proteasome mediated degradation of protein depends on the functioning of these three DUBs. While USP14 and UCH37 antagonize degradation, RPN11 seems to promote substrate degradation (Lee et al., 2010; De Poot et al., 2017). If a substrate related to mitophagy is shared between these DUBs, how they interplay and decide the fate of the protein requires further independent study. However, if USP14 mediated enhanced proteasome activity and subsequently mitophagy is to be investigated, a simpler way can be followed. The effect of the inhibitor on proteasome activity in specific areas can be monitored by using synthetic flourogenic substrates. In this regard, it should also be kept in mind that many of these substrates enter the core of proteasome complex quite freely and thus may minimize the quantitative differences between the groups.

To simplify our point of view, we refer to Figure 2E. Here, we presented a few of the considerations that should be determined beforehand. We also represented a few anticipated outcomes concerning mitophagy after USP14 inhibition, based on two situations: (+) means high and (−) mean low, prior to inhibitor administration. The current understanding suggests that if age and area associated USP14 levels is already at a low level, USP14 inhibition might not be able to enhance mitophagy further. The same can be expected if PHB2 level is low. So an unaltered or higher level of USP14 and PHB2 is expected for inhibitor mediated enhanced mitophagy. If a particular model of neurodegenerative disorder downregulates USP14 or negatively impacts the accessibility of the inhibitor to the area, that should also negatively impact the outcome after the administration of the inhibitor.

## Discussion

Aging and autophagy are quite inversely related. This phenomenon contributes to many of the age-associated neuronal complicacies (Simonsen et al., 2008; Pyo et al., 2013; Carnio et al., 2014; Hansen et al., 2018). However, goal of the current perspective is not to highlight age-related autophagy or proteasome functioning. Instead, we wanted to point out a few of the important aspects for evaluating USP14 as a therapeutic target, which might act as an upstream effector of mitophagy in rat brain. This perspective highlights mostly one aspect: there could be age and disorder specific alterations in USP14 protein levels which might impact the outcome. As in this case, an increase in USP14 is found mostly in SN, it can be advocated that it might be a key target for therapy development, as far as PD related anomaly in mitophagy is concerned.

Though USP14 is more pronounced for its therapeutic potential in cancer (Shinji et al., 2006; Liao et al., 2018; Han et al., 2019), its role in neurodegenerative disorders is also known (Boselli et al., 2017; Min et al., 2017; Chakraborty et al., 2018). It is well accepted that the influence of USP14 on proteasome complex activity is inversely correlated (Lee et al., 2010; Kim and Goldberg, 2017). Because in many of the neurodegenerative disorders proteasome complex is known to be inhibited and one of the causative factors for protein aggregate formation (Keller et al., 2000; Mcnaught et al., 2001, 2002; Diaz-Hernandez et al., 2006; Thibaudeau et al., 2018) inhibitors of USP14 has been proposed as curative agents (Lee et al., 2010; Boselli et al., 2017). Whether or not the available inhibitor – IU1 or its derivative is toxic for neuronal cells, is a matter of debate as reports indicate both the ways for cultured neurons (Boselli et al., 2017; Kiprowska et al., 2017). Boselli et al. (2017) did not find any toxicity with IU1-47 (a more potent inhibitor of USP14 than IU1) and demonstrated that it can enhance Tau degradation. Kiprowska et al. (2017), however, showed that IU1 decreases neuronal survivability and inhibits ETC complex I in isolated mitochondrial fraction. It should be noted that culture conditions for cortical neurons are important in this regard as it can influence the neuronal toxicity of IU1 or its derivatives.

How USP14 can influence autophagy is yet to be fully characterized. Though the study by Xu et al. (2016) suggests that USP14 inhibition leads to stabilization of K63 ubiquitination of Beclin1 and thus enhances autophagy (Xu et al., 2016) whether long term inhibition will also lead to the same or not, might be a matter of discussion. Long term enhancement of proteasome functioning might induce degradation of some of the protein members of autophagy machinery and thus might have a negative feedback loop. This feedback loop between autophagy and UPS is unavoidable and the duration of USP14 inhibition may decide the outcome. Precautions should be taken to utilize this window period and avoid such paradoxes. In our opinion determining and utilization of the therapeutic window period is vital for exploring the protective effects of USP14 inhibition. Here, we would like to highlight that USP14 is required for maintaining the free ubiquitin pool and it is vital for normal neuronal functioning and plasticity (Anderson et al., 2005; Vaden et al., 2015b). USP14 deficient mice exhibit post-natal lethality, motor incoordination and defects in neuromuscular junction, which can be rescued by overexpression of the protein (Crimmins et al., 2006; Chen et al., 2009; Vaden et al., 2015b). USP14 inhibition in amygdala also results in impairment of long term potentiation against fear conditioning (Jarome et al., 2013). So, chronic USP14 inhibition might lead to unavoidable damage to peripheral and central nervous system in rodents. If USP14 mediated mitophagy is to be targeted in rodent models of neurodegenerative disorders, we propose intermittent USP14 inhibition for exploring the therapeutic aspects. In our view, this will enhance the chances of mitochondrial rejuvenation with minimal toxic effects on neurophysiology. The dose and duration of the inhibition have to be standardized in a disease-specific manner. It has to be mentioned here, Vaden et al. (2015a) demonstrated that acute, intermittent administration of IU1 does not affect structure and arborization of neuronal endplates as such, but reduce miniature endplate current frequency and enhance AChR-γ expression in gastrocnemius muscle. The study further demonstrated that USP14 deficiency mediated complicacies in neuromuscular junction is executed via c-Jun N- terminal kinase (JNK) signaling. JNK inhibition rescued motor dysfunction and abnormalities in synaptic structures caused by USP14 deficiency. So incorporating JNK inhibitors in the treatment paradigm might minimize the anticipated impairments caused by USP14 inhibition and extend therapeutic window period in rodent models of neurodegeneration.

How USP14 increases immediately in response to ETC complex I blockade is not clear. Dopamine itself might not be a factor for this enhancement, as VTA did not show any alterations after rotenone treatment. Whether or not this effect persists in long term treatment is a matter of further study. However, the implications of such site-specific increase could be vast. First of all, this might lead to a decrease in proteasome activity and in turn can lead to early accumulation of protein oligomers, which are known to act as the “seeds” for further protein aggregation (Gregori et al., 1995; Lindersson et al., 2004; Diaz-Hernandez et al., 2006). Secondly, this increase in USP14 might also lead to reduced levels of mitophagy. The most important point here we state is the increase in USP14 level is SN specific. Investigations are warranted to determine whether or not this is the initiation point for decreased UPS activity and reduced mitophagy in PD.

## Materials and Methods

### Materials

All the chemicals are analytical grade and purchased from Sigma Aldrich Chemicals Pvt Ltd. or Sisco Research Laboratories Pvt. Ltd. (SRL, India) unless otherwise specified. Reagents were prepared and stored according to the manufacturer’s guidelines.

### Animal Treatment

Animal experimentations were carried out as per national guidelines on the “Care and Use of Animals in Scientific Research,” formed by Committee for the Purpose of Control and Supervision of Experiments on Animals (CPCSEA), Animal Welfare Division, Ministry of Environment and Forests, Govt. of India. The protocol was accepted by animal ethics committee of CSIR-Indian Institute of Chemical Biology, Kolkata, India. Sprague Dawley male rats (1 month, 6–7 and 10–12 months old) were kept in the animal house (22 ± 2°C, 60 ± 5% humidity, with 12 h light-dark cycle). Food and water were provided *ad libitum*. 3-NP was made freshly before each injection in saline (0.85% NaCl) and the pH was adjusted to 7.4 by 5.0 N NaOH. Rats were treated with 20 mg/kg (i.p.) once daily, for 2 days. Rotenone was dissolved in 30:70 DMSO and mineral oil and 1 mg/kg (i.p.) dose was administered for 2 days.

### Immunoblotting

In brief, isolated brain regions were homogenized in ice cold radio immunoprecipitation buffer (RIPA, 50 mM Tris HCl containing 1 mM EDTA, 150 mM NaCl, 1% Nonidet p-40, 0.25% sodium deoxycholate; pH 7.5) supplemented with protease inhibitors (Invitrogen). The lysate was kept on ice for 30 min and centrifuged at 10,000 × *g* for 10 min at 4°C and the supernatant was collected. 25–40 μg protein was separated by 10% polyacrylamide SDS gels and transferred to PVDF membrane. Blocking and antibody dilution was done in 5% or 2.5% skimmed milk, respectively. The following antibodies were used: anti-Actin (1:2,000; Santa Cruz Biotechnology), anti-PHB2 (1:3,000; Sigma), anti-PHB1 (1:1000; Abcam) and Anti USP14 (1: 3000, Abcam). Appropriate HRP tagged secondary antibodies (rabbit or mouse, 1:2000) were procured from Bangalore Genei Private Limited (India).

Protein bands were detected using chemiluminescence substrate (Sigma) and images were captured in a chemidoc instrument (Biorad). Band intensities were measured by ImageJ and were normalized by respective Actin band intensity.

### Statistics

We used two tailed student’s *t*-test and one way ANOVA followed by Newman Keul’s multiple comparison test for statistical significance. In all cases, results are provided as mean ± S.E.M., and *p* ≤ 0.05 was considered as significant.

## Data Availability Statement

The raw data supporting the conclusions of this article will be made available by the authors, without undue reservation.

## Ethics Statement

The animal study was reviewed and approved by the animal ethics committee and experimentations were carried out in accordance with national guidelines on the “Care and Use of Animals in Scientific Research,” formed by Committee for the Purpose of Control and Supervision of Experiments on Animals (CPCSEA), Animal Welfare Division, Ministry of Environment and Forests, Govt. of India. The protocol was accepted by animal ethics Committee of CSIR-Indian Institute of Chemical Biology, Kolkata, India.

## Author Contributions

JC, CB, and MR performed the experiments with young, adult, and mid-aged rats, as well as with the rotenone treatment. RM performed the experiments with 3-NP treatment. JC designed the experiments and analyzed the data. CB, MR, and JC wrote the perspective.

## Conflict of Interest

The authors declare that the research was conducted in the absence of any commercial or financial relationships that could be construed as a potential conflict of interest.
