# Corrigendum: USP14 as a therapeutic target against neurodegeneration: a rat brain perspective

**DOI:** 10.3389/fcell.2023.1244890

**Published:** 2023-07-20

**Authors:** Chayan Banerjee, Moumita Roy, Rupsha Mondal, Joy Chakraborty

**Affiliations:** ^1^ Department of Cell Biology and Physiology, CSIR-Indian Institute of Chemical Biology-TRUE, Kolkata, India; ^2^ CSIR-Indian Institute of Chemical Biology, Kolkata, India; ^3^ Academy of Scientific and Innovative Research (AcSIR), Ghaziabad, India

**Keywords:** USP14, Prohibitin2, mitophagy, neurodegeneration, Parkinson’s disease, substantia nigra, rotenone, 3-nitropropionic acid

In the published article, there was an error in **affiliation 3**. Instead of “Academy of Scientific and Innovative Research (AcSIR), CSIR- Human Resource Development Centre, Ghaziabad, India”, it should be “Academy of Scientific and Innovative Research (AcSIR), Ghaziabad, India”.

The authors apologize for this error and state that this does not change the scientific conclusions of the article in any way. The original article has been updated.

